# Marginal Integrity of Cervical Restorations with Caries-Affected Dentinal Walls: Effect of Contamination with Hemostatic Agents

**Published:** 2018-07

**Authors:** Maryam Khoroush, Fatemeh Keshani, Mehdi Esmaeili, Moeen Hosseini Shirazi

**Affiliations:** 1 Professor of Restorative Dentistry, Dental Materials Research Center, Torabinejad Dental Research Center, Department of Operative Dentistry, School of Dentistry, Isfahan University of Medical Sciences, Isfahan, Iran; 2 Assistant Professor of Restorative Dentistry, Dental Materials Research Center, Department of Restorative Dentistry, School of Dentistry, Isfahan University of Medical Sciences, Isfahan, Iran; 3 Dentist, Private Practice, Isfahan, Iran; 4 Assistant Professor, Department of Prosthodontics and Implantology, School of Dentistry, Babol University of Medical Sciences, Babol, Iran

**Keywords:** Hemostatics, Dental Leakage, Dentin-Bonding Agents

## Abstract

**Objectives:**

The aim of this study was to compare the microleakage in normal and caries-affected dentin (CAD) and to investigate the effect of three hemostatic agents on the microleakage of Class V composite resin restorations in CAD.

**Materials and Methods:**

Ninety-six Class V non-beveled cavities were prepared in 48 third molars at 1 mm below the cementoenamel junction (CEJ) in the cervical margin with the occlusogingival size of 2 mm, mesiodistal dimension of 3 mm, and a depth of 1.5 mm. Next, the teeth were divided into 8 groups (n=12): G1-4 included normal dentin (N) substrate, while G5-8 were exposed to mineralization/demineralization cycles to produce CAD substrate. Groups 1 and 5 were the controls. ViscoStat was used in groups 2 and 6, ViscoStat Clear was used in groups 3 and 7, while trichloroacetic acid (TCA) was used in groups 4 and 8. The cavities were restored with composite resin. The samples were sectioned after thermocycling and immersion in 2% fuchsin for 24 hours. The degree of dye penetration was evaluated under a stereomicroscope at 40× magnification. Data were evaluated using Kruskal-Wallis and Mann-U-Whitney tests in SPSS 15 software (α=0.05).

**Results:**

Significant differences were recorded on the mean microleakage of different groups (P=0.047). There was a significant difference in the mean dentinal microleakage between N and CAD groups (P=0.014). The dentinal microleakage in group 3 was significantly higher than that in groups 4 to 8.

**Conclusions:**

According to the results, CAD showed less microleakage in comparison with intact dentin. ViscoStat Clear caused a greater microleakage than did ViscoStat or TCA.

## INTRODUCTION

Despite recent advances in restorative dentistry, microleakage and the consequent discoloration of the margins and postoperative sensitivity result in the failure of composite resin restorations [1]. In most studies, microleakage is characterized as the primary reason for secondary dental caries, pulp inflammation, and necrosis [2, 3]. Microleakage is defined as the passage of small amounts of fluids containing microorganisms, molecules, and ions, which are clinically undetectable, through microscopic spaces between a dental restoration and the adjacent surface of the cavity preparation [3]. There are several reasons for microleakage in composite restorations including polymerization shrinkage, a difference between the expansion coefficients of resin and dental tissue, lack of a self-sealing mechanism, and occlusal loading [4]. Lack of an efficient bonding between composite resins and dental surfaces is one of the major reasons for microleakage. Dentin bonding efficiency depends on the appropriate interaction between the resin and dentinal collagen fibers. Contaminants such as blood and the gingival crevicular fluid (GCF) result in a decrease in the bonding efficacy by interfering with the penetration of resin tags into collagen fibers [5, 6]. In cervical restorations with gingival margins below the cementoenamel junction (CEJ), isolation is not always possible. Usually, bleeding occurs during these restorative procedures following trauma to the gingiva, or the GCF contaminates the prepared surfaces [4, 7].

One method to control bleeding and contamination by the GCF is to use hemostatic agents [8]. Use of hemostatic agents has raised the question whether or not contamination with these materials affects bonding to the dental surface [9]. Hemostatic agents such as 25% aluminum chloride, 20% ferric sulfate, and 35% trichloroacetic acid (TCA) are frequently used in dentistry [9, 10]. TCA is a very acidic chemical agent with a pH of 1.0, which has often been used for decalcification and fixation purposes in microscopic studies. It has also been used for precipitating proteins and as a cauterizing agent in medical procedures [8, 11].

Recently, the self-etching adhesion strategy, in which the substrate is not etched separately, is predominantly used due to its simple application. This strategy simplifies the bonding as it does not require etching and it reduces the time needed for restoration [12]. In addition, this system is not as technique-sensitive as etch-and-rinse strategies and prevents the creation of a large demineralized region [13]. However, considering the effect of the smear layer on the bonding of self-etch systems, removing the smear layer by hemostatic agents may affect the bonding mechanism of these systems [14–16].

The methods involving preparation of large cavities are replaced by more conservative approaches to save tooth structure as much as possible. In these techniques, only the outer layer of carious dentin, which is infected and necrotic, is removed, while the inner layer, which is called the affected dentin, is left in place. This affected dentin is demineralized but has the potential to be remineralized [17]; adhesives bond to this affected dentin during composite resin restoration process. The application of hemostatic agents to the above-mentioned cavities with caries-affected dentinal walls can modify the bond strength of restorative materials to the cavity surfaces [18–21].

Considering the conservative approach to preserve the affected dentin and the use of self-etch adhesive systems, the aim of this study was to compare microleakage in normal dentin (N) and caries-affected dentin (CAD) and to investigate the effect of hemostatic agents (ViscoStat, ViscoStat Clear, and TCA) on the microleakage of Class V composite resin restorations in N and CAD. The null hypothesis was that the microleakage in N and CAD are the same, and also, hemostatic agents have no effect on the microleakage of restorations in CAD.

## MATERIALS AND METHODS

In this experimental study, 48 intact human third molars from the upper and lower jaws were selected, cleaned and rinsed with brushes and diluted pumice, and stored in 0.2% thymol solution before being used. Then, the teeth were embedded in a self-cured acrylic resin (Unifast III, GC Corp., Tokyo, Japan) and were kept in water until the complete curing to control the thermal effects.

A total of 96 Class V non-beveled cavities were prepared in the buccal and lingual surfaces of the teeth using a diamond bur (Hi-Di®, Dentsply Sirona, London, UK) mounted on a high-speed handpiece at 1 mm below the CEJ in the cervical margin, with the occlusogingival size of 2 mm, mesiodistal dimension of 3 mm, and a depth of 1.5 mm. Afterwards, these 48 cervical cavities were exposed to mineralization/demineralization cycles. These cycles included 3 hours of immersion in a demineralizing solution followed by 45 hours of immersion in a mineralizing solution. The specimens were submitted to 8 mineralization/demineralization cycles, each taking 48 hours. The demineralizing and mineralizing solutions were refreshed after the 4th cycle and after each cycle, respectively [22]. The composition of the demineralizing and mineralizing solutions are given in Table 1.

**Table 1.** The composition of the demineralizing/remineralizing solutions

**Demineralizing solution (pH=4.5)**	2.2 mM calcium (CaCl2)
	2.2 mM phosphate (NaH2PO4)
	0.05M sodium acetate (C2H3NaO2)
	0.05M acetic acid (CH3COOH)
	1 ppm fluoride (NaF)
**Remineralizing solution (pH=7)**	1.5 mM calcium (CaCl2)
	0.9 mM phosphate (NaH2PO4)
	0.15M KCl
	0.1M Tris buffer (C4H11NO3)
	10 ppm fluoride (NaF)

Ppm=Part per million, NaF=Sodium fluoride, KCl=Potassium chloride

Next, the prepared teeth were divided into 8 groups (n=12; Table 2).

**Table 2.** Groups under study in terms of dentin type and hemostatic agents

**Dentin type**	**Group number**	**Group name**	**Hemostatic agent**
**N**	1	N/-	-
	2	N/Vis	ViscoStat
	3	N/Vis.Clear	ViscoStat Clear
	4	N/TCA	TCA
**CAD**	5	CAD/-	-
	6	CAD/Vis	ViscoStat
	7	CAD/Vis.Clear	ViscoStat Clear
	8	CAD/TCA	TCA

N=Intact dentin, CAD=Caries-Affected Dentin, TCA= Trichloroacetic acid, Vis=ViscoStat, Vis.Clear=ViscoStat Clear

In groups N (intact dentin) and CAD/-, which are the control groups, no hemostatic agents were used. In groups N/Vis and CAD/Vis, ViscoStat (Ultradent Products Inc., South Jordan, UT, USA) was applied to the cavity walls for 2 minutes according to the manufacturer’s instructions. Then, the teeth were rinsed with water spray for 30 seconds and were dried with oil-free air. In groups N/Vis.Clear and CAD/Vis.Clear, ViscoStat Clear (Ultradent Products Inc., South Jordan, UT, USA) and in groups N/TCA and CAD/TCA, TCA were used in the same way as groups N/Vis and CAD/Vis. ViscoStat and ViscoStat Clear consist of 20% ferric sulfate and 25% aluminum chloride, respectively. Subsequently, a mild two-step self-etch adhesive (Clearfil SE Bond, Kuraray, Okayama, Japan) was used according to the manufacturer’s instructions as follows: applying the primer using a saturated brush tip for 20 minutes, drying with mild air, applying the adhesive and then light curing for 10 seconds. The cavities were restored with the A3 shade of a light-cured composite resin (APX, Kuraray, Tokyo, Japan) which was cured for 40 seconds using a light-curing unit (Coltolux 50, Mod. C7950, Coltene/Whaledent Inc., Cuyahoga Falls, OH, USA). The restorations were finished using fine diamond burs, polished with polishing discs (3M ESPE, St. Paul, MN, USA), and were kept at 37°C for 24 hours. After 1000 thermal cycles, the teeth were stored in 2% fuchsin solution for 24 hours and were then sectioned buccolingually parallel to the longitudinal axis of the tooth using a 0.3-mm-thick diamond-coated cutting disc (Buehler® IsoMet® Diamond Wafering Blade, No 11-4254, Düsseldorf, Germany). Dye penetration in the samples was investigated under a stereomicroscope (SMP-200, HP, Palo Alto, CA, USA) at 40× magnification. The specimens were scored based on the degree of dye penetration into all cavity margins using the following scoring system [22]:

0: no penetration
1: dye penetration up to 1/3 of the cavity depth
2: dye penetration between 1/3 and 2/3 of the cavity depth
3: dye penetration more than 2/3 of the cavity depth towards the pulp

Data were analyzed with Kruskal-Wallis and Mann-U-Whitney tests using SPSS for Windows (SPSS Inc., Chicago, IL, USA) Release 15.0.0 (α=0.05).

## RESULTS

Table 3 shows the numbers related to microleakage at dentinal margins.

**Table 3.** Frequencies and percentages of microleakage at dentinal margins

**Groups (n=12)**	**microleakage at dentinal margins N(%)**			

	**0**	**1**	**2**	**3**
**N/-**	0(0)	5(41.7)	1(8.3)	6(50)
**N/Vis**	0(0)	3(25)	4(33.3)	5(41.7)
**N/Vis.Clear**	0(0)	1(8.3)	3(25)	8(66.7)
**N/TCA**	0(0)	5(41.7)	4(33.3)	3(25)
**CAD/-**	1(8.3)	4(33.3)	6(50)	1(8.3)
**CAD/Vis**	0(0)	4(33.3)	5(41.7)	3(25)
**CAD/Vis.Clear**	1(8.3)	5(41.7)	2(16.7)	4(33.3)
**CAD/TCA**	0(0)	4(33.3)	8(66.7)	0(0)

N=Intact dentin, CAD=Caries-Affected Dentin, TCA= Trichloroacetic acid, Vis=ViscoStat, Vis.Clear=ViscoStat Clear

**Fig. 1.**
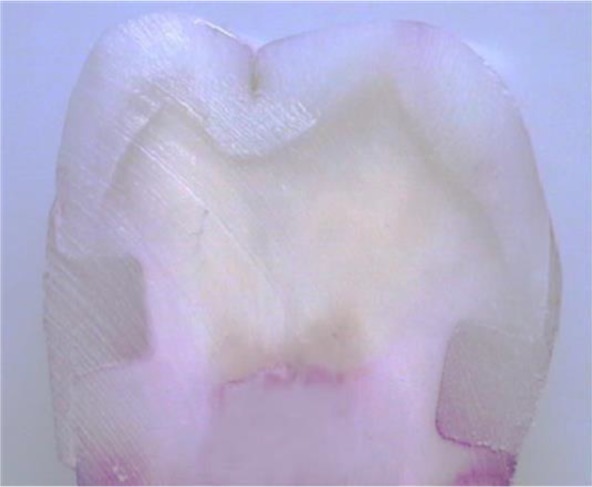
Cross-section of a sample with dye penetration score of 3 at dentinal margins

Figure 1 shows the cross-section of a sample with dye penetration score of 3 at dentinal margins. According to Kruskal-Wallis test, the mean microleakage in different groups showed significant differences (P=0.047).

Moreover, significant differences were observed in the dentinal microleakage between the normal groups and the demineralized groups (P=0.014). Mann-U-Whitney test was performed for pairwise comparisons of the dentinal microleakage in each group. Table 4 shows the groups for which significant differences were observed in dentinal microleakage with the corresponding P-values. The dentinal microleakage in N/Vis.Clear group was shown to be significantly higher compared to groups under demineralization and also compared to N/TCA group.

**Table 4.** Comparing the dentinal microleakage in different groups

	**N/TCA**	**CAD/-**	**CAD/Vis**	**CAD/Vis.Clear**	**CAD/TCA**
**N/Vis.Clear**	0.028	0.004	0.037	0.042	0.002
**N/Vis**	0.018	0.0031	0.002	0.012	0.016

N=Intact dentin, CAD=Caries-Affected Dentin, TCA= Trichloroacetic acid, Vis=ViscoStat, Vis.Clear=ViscoStat Clear

Data were grouped according to hemostatic agents as variables, and Kruskal-Wallis test showed no significant differences between the dentinal microleakage of the control groups and that of the groups of ViscoStat, ViscoStat Clear, and TCA (P=0.27).

## DISCUSSION

It is essential to avoid any contamination on the surface of the cavity before applying composite resin and adhesive systems to achieve a strong and durable adhesion. However, application of a rubber dam is difficult in most cervical lesions, which makes contamination by blood or saliva inevitable [19]. One way to control bleeding is to use local hemostatic agents. Due to the fewer number of components and the fewer number of steps in their application, self-tech adhesive systems can be used in a shorter time, reducing the odds of blood contamination [6, 10]. In addition, they avoid accidental contact of gingival margins of the cavity with phosphoric acid during etching and rinsing [10]. Kumar et al [4] evaluated the effect of various surface contaminants on the microleakage of two different generations of adhesive systems (Single Bond and iBond), reporting that when cavities were exposed to the hemostatic agent (ViscoStat), Single Bond resulted in a significant microleakage at the gingival margin with no significant microleakage at the enamel margin when compared to the no contamination scenario, whereas iBond exhibited a significant microleakage at both margins.

Kimmes et al [5] reported that ViscoStat Plus (22% ferrous chloride) and ViscoStat (20% ferrous sulfate) hemostatic agents did not affect the shear bond strength with the use of total-etch adhesive systems.

Rocha et al [22] investigated the shear bond strength of luting agents to CAD and showed that sound dentin exhibited a higher hardness than CAD. Both resin cements used in the cited study showed a higher shear bond strength to sound dentin than to CAD [22].

Khoroushi and Tavasoli [8] investigated the effect of TCA as etching and hemostatic agents on the enamel surface texture and the bond strength of composite resins to enamel. Their results demonstrated that TCA improved the bond strength of composite resin to enamel [8]. A two-step self-etch adhesive system was used in the current study. Considering the adhesive bonds to the affected dentin in recent conservative restorations, two substrates of sound dentin and CAD were considered. In the current study, dentinal microleakage was shown to be significantly higher in groups with sound dentin substrate compared to the groups with CAD substrate (P=0.014). Unlike the current study, some studies have shown the lower shear bond strength of etch-and-rinse adhesives to CAD than to sound dentin [22–24]. According to some studies, specific changes have been observed in the mineral contents of CAD. In particular, intertubular dentin has been shown to have a lower mineral content compared to normal dentin; thus, due to the reduced resistance to the applied acid, dentinal tubules are eliminated [23, 24].

Blockage and elimination of dentinal tubules may interfere with resin infiltration [22, 25, 26]. On the contrary, the lower mineral contents of intertubular dentin in CAD allows deeper etching in this substrate [22, 26, 27]. To explain the lower shear bond strength to CAD, researchers have reported the existence of crystals in dentinal tubules, which limit the expansion of resin tags [22, 23]. Regardless of the fact that CAD is artificially made and does not have crystals, resin tags have a weak correlation with bond strength. Therefore, deeper demineralization of phosphoric acid in CAD, which is more porous, can explain this observation. In CAD, inconsistency between the demineralization depth and infiltration of adhesive resin results in the formation of a thicker layer of unsupported collagen at the base of the hybrid layer with no minerals or adhesives. This mineral-free layer acts as a weak component during the shear test and results in a decrease in bond strength [22]. However, the mild self-etch adhesive used in the current study was not capable of deep demineralization compared to phosphoric acid. This reduces the possibility of formation of that weak component at the base of the hybrid layer. On the other hand, the lower mineral content of intertubular dentin in CAD and its higher porosity allows deeper etching in this substrate compared to sound dentin and may cause less microleakage [24]. Nonetheless, noting that hemostatic agents are acidic and can affect demineralization, further studies are recommended on this subject.

The current study showed a significantly higher microleakage at dentinal margins in N/Vis.Clear group compared to other groups with CAD substrate and N/TCA group. Consistent with this study, noting the aluminum chloride content of ViscoStat Clear, Mohammadi et al [19] showed that aluminum chloride (Hemostop, Dentsply, Argentina) led to a significant increase in microleakage at gingival margins with all-in-one adhesive resin. Surface contamination with aluminum chloride decreases demineralization by the self-etch adhesive system, which might be explained by substitution of calcium in hydroxyapatite by the hemostatic agent, producing an insoluble compound. This results in the limitation of demineralization in self-etch systems with weak acidity [26]. In contrast, Kuphasuk et al [18] investigated the effect of aluminum chloride hemostatic agent on total-etch systems and reported no difference between the bond strength to normal dentin and that of contaminated dentin. This difference might be due to the application of phosphoric acid with high acidity. Furthermore, a decrease in the sealing capability and an increase in microleakage might be related to the removal of the smear layer, which affects the bonding mechanism in self-etch systems [17, 18]. Previous studies have shown that hemostatic agents are highly acidic, and their pH varies between 0.7 and 3.0. Aluminum chloride at a concentration of 20–25% has been shown to demineralize dentinal surfaces with different scales and patterns. Furthermore, demineralization has been observed to some extent on the dentin surface contaminated with 21.3% aluminum chloride for only 5 minutes, and the smear layer was totally removed in this case [19,20]. The results of using different hemostatic agents in the present study were in line with the results of previous studies in case of removing the smear layer, but the various patterns may be due to the use of different materials and test conditions.

Finally, as this study was conducted in the laboratory environment and since evaluation was not possible in the presence of blood or GCF contaminations, more comprehensive clinical evaluations with larger numbers of specimens are recommended in this regard.

## CONCLUSION

Considering the limitations of the current research, the following conclusions can be made: Microleakage in dentinal margins was shown to be significantly higher in the groups with sound dentin substrate compared to the groups with CAD substrate; this confirms the advantages of conservative dentistry and maximum maintenance of dental structures.

ViscoStat was shown to cause a higher dentinal microleakage than TCA; however, ViscoStat Clear triggered the highest dentinal microleakage compared to the other hemostatic agents.
